# Multimorbidity patterns and their associated factors among patients with type 2 diabetes in China: A hospital-based observational study

**DOI:** 10.1016/j.heliyon.2025.e42905

**Published:** 2025-02-21

**Authors:** Jin Li, Hou Hou, Yong Zhang, Jing Li

**Affiliations:** a Department of Neurology, Chu Hsien-I Memorial Hospital & Tianjin Institute of Endocrinology, Tianjin Medical University, Tianjin, 300134, China; b NHC Key Laboratory of Hormones and Development, Tianjin Key Laboratory of Metabolic Diseases, Tianjin, 300134, China; c Department of Rheumatology and Immunology, Tianjin Medical University General Hospital, Tianjin, 300070, China

**Keywords:** Type 2 diabetes, Multimorbidity, Risk factors, Latent class analysis

## Abstract

**Introduction:**

Most patients with type 2 diabetes mellitus (T2DM) suffer from multimorbidity, but the multimorbidity patterns were not well understood. We aimed to ascertain the different multimorbidity patterns among T2DM patients and to explore the corresponding associated factors.

**Method:**

The study included 3403 T2DM patients from Tianjin, China. Multimorbidity (including dyslipidemia, hypertension, hyperuricemia, cardiovascular diseases, chronic kidney disease, and chronic liver disease) was ascertained through medical records. Data were analyzed using latent class analysis and multi-nominal logistic regression.

**Results:**

The leading 3 morbidity were dyslipidemia, cardiovascular diseases, and hypertension; and 16.9 % had multimorbidity. Four unique patterns were ascertained: multimorbidity-free, T2DM with cardiovascular diseases mainly, T2DM with dyslipidemia mainly, and T2DM with hypertension mainly. In the subsample of 1779 patients, greater BMI and LDL, and non-use of insulin were associated factors for cardiovascular disease patterns; older age, higher HDL, lower LDL, and insulin use for dyslipidemia patterns; male sex, higher TG and LDL, and non-use of insulin for hypertension patterns.

**Conclusions:**

Dyslipidemia, cardiovascular diseases, and hypertension were the most common chronic conditions among T2DM patients. Four groups of T2DM-associated multimorbidity patterns were identified, and different patterns had varying associated factors. Our research findings highlight the significance of formulating personalized nursing measures for patients with different T2DM multimorbidity patterns.

## Highlights

• The leading 3 morbidity of type 2 diabetes mellitus (T2DM) were dyslipidemia, cardiovascular diseases, and hypertension.
• Four different patterns of T2DM were ascertained including multimorbidity-free, T2DM with cardiovascular diseases mainly, T2DM with dyslipidemia mainly, and T2DM with hypertension mainly.
• Different multimorbidity patterns had varying influencing factors.

## 1 Introduction

China has the largest elderly population and the fastest aging rate worldwide [1], and the incidence and burden of diabetes are increasing [2]. The Global Burden of Disease, Injury, and Risk Factors Study estimates that in 2019, there will be about 92 million people with diabetes in China, resulting in about 1.55 million deaths [3]. The aging population not only promotes type 2 diabetes mellitus (T2DM) but also contributes to its associated complications [4]. Recent studies of the global disease burden have shown that various chronic diseases have increased annually [5,6]. Multimorbidity (defined as the coexistence of ≥2 chronic conditions) is a growing problem not only for high-income countries but also for low- and middle-income countries, which account for 77 % of global NCD deaths, 85 % of which are premature [7]. Notably, the majority (up to 90 %) of patients diagnosed with T2DM suffer from multimorbidity, with 23 % having ≥4 chronic conditions [8]. The multimorbidity of various chronic diseases among T2DM patients has been associated with reduced quality of life, impaired functional status, and increased burden on limited healthcare resources [9]. Thus, controlling these chronic diseases is an effective way to reduce global and national health inequities [10].

The multimorbidity of patients with T2DM highlights their unique healthcare requirements and tailored management strategies. Adopting a patient-focused approach to multimorbidity management requires a comprehensive understanding of patterns of comorbidities [11]. However, current evidence on the multimorbidity patterns in patients with T2DM is limited [[11], [12], [13], [14], [15]], especially in developing countries, and the differences in the risk factor profiles for different patterns are poorly understood. Most of the T2DM multimorbidity studies have utilized simple descriptive [8,14], hierarchical clustering [12,13], or K-means methods [15], which fail to take into account the complex associations among the diseases and lack integrity, and thus are inferior to latent class analysis (LCA) in the identification of multimorbidity patterns [16]. Moreover, only one study from India found that older age, female sex, use of insulin, and/or oral medications were associated with multimorbidity patterns in patients with T2DM [14]. A Chinese data-based study revealed multimorbidity networks associated with T2DM, but the specific influencing factors of these different network patterns were not further investigated [11]. To date, the multimorbidity patterns and their specific associated factors have not been understood in Chinese patients with T2DM.

In the present study, we aimed to 1) ascertain the different multimorbidity patterns among patients with type 2 diabetes in China, and 2) examine the associated factors of diabetic multimorbidity patterns in a clinical population-based cross-sectional study from China.

## 2 Materials and methods

### 2.1 Study design, setting, and participants

**Fig. 1:**
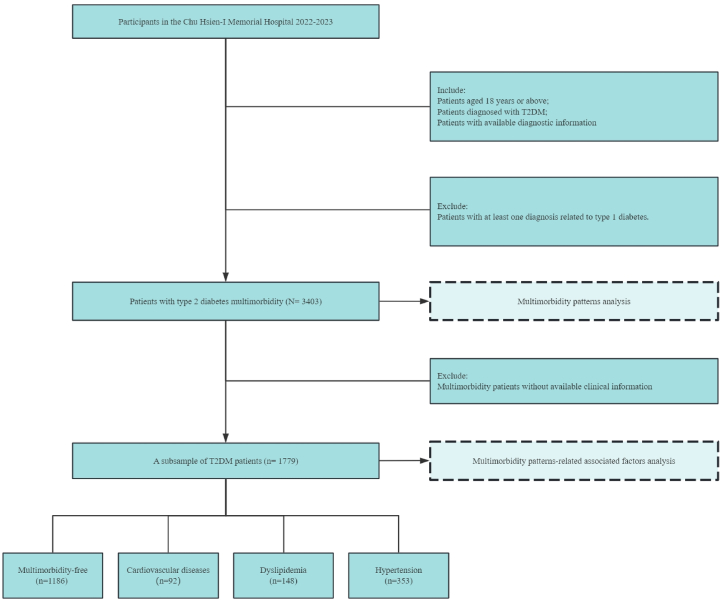
Participants included in the analysis.

The study was conducted as a single-center retrospective analysis in Tianjin, China (a tertiary hospital). The study population comprised patients who had been diagnosed with T2DM aged 18+ years from 2022 to 2023. The participants' records were retrieved from the hospital's electronic health record system. Patients aged 18 years or above, diagnosed with T2DM, and with available diagnostic information were included in the analysis. In addition, patients who had at least one diagnosis related to type 1 diabetes were excluded from the current study. Finally, a total of 3403 T2DM patients were included in the multimorbidity patterns analysis. Among them, a subsample of 1779 T2DM patients with available clinical information was further included in the multimorbidity patterns-associated factors analysis (Fig. 1).

### 2.2 Data collection

All participants underwent a comprehensive physical examination and clinical evaluation. The information on demographic characteristics (such as age and sex), and lifestyle factors (like smoking status and alcohol consumption) were collected. This information was retrieved from the participants' medical records and, in some cases, through interviews with the patients or their legal guardians. Data collection was carried out by experienced clinical nurses, who underwent unified training within the team before data extraction. For any inconsistencies and logical issues in the data, a double-check was performed by two individuals, and a final decision was made by an independent third party.

### 2.3 Assessment of multimorbidity patterns

Multimorbidity was identified through the inpatient registry system and the patient's self-reported information. Information on chronic diseases including dyslipidemia (E78.0, E78.2), hypertension (I10, I15.0–2, I15.8–9), hyperuricemia (M10), cardiovascular diseases (I20-I22, I48), chronic kidney disease (N18), and chronic liver disease (B18) was recorded and used for subsequent multimorbidity pattern analysis.

### 2.4 Assessment of covariates

The study collected information on several potential covariates, including demographics (age, sex, smoking status, and alcohol consumption status), clinical characteristics (body mass index [BMI], glycosylated hemoglobin [HbA1c], total cholesterol [TC], triglyceride [TG], high-density lipoprotein cholesterol [HDL], low-density lipoprotein cholesterol [LDL], blood glucose, and family history), and treatment patterns (use of antidiabetic drugs and insulin). Fasting venous plasma samples were collected and tested at the time of enrollment. All sample processing followed the international standard procedures. HbA1c was detected by high-performance liquid chromatography.TC, TG, and HDL were measured using the enzymatic colorimetric method. LDL was calculated based on the Friedewald equation. Blood glucose was determined by the glucose oxidase method.

### 2.5 Statistical analysis

#### 2.5.1 Construct the multimorbidity patterns

Latent class analysis (LCA) was used to group patients into distinct multimorbidity classes, which can identify hidden clusters by grouping multiple observed variables (i.e. chronic diseases) into a latent variable with mutually exclusive latent classes (i.e. the multimorbidity patterns), which could appropriately capture multiple T2DM-related morbidities and their complex interplays simultaneously. LCA was performed in patients with at least one morbidity. To find the optimal fitting model for the current data, LCA models with one to five latent classes were conducted. Model fit was assessed using metrics such as the Akaike Information Criterion (AIC), Bayesian Information Criterion (BIC), maximum log-likelihood, G2, and entropy, with smaller values indicating better model fit. The conditional probability of the LCA model was used to name the final multimorbidity patterns.

#### 2.5.2 Ascertain associated factors of multimorbidity patterns

Baseline characteristics of the study population were presented as number (%) for categorical variables and mean ± standard for continuous variables. Baseline characteristics of participants were summarized across multimorbidity patterns, and differences were tested by using the Kruskal-Wallis H test for continuous variables or the chi-square test for categorical variables. Multinomial logistic regression was used to estimate odds ratios (ORs) with 95 % confidence intervals (CIs) for the multimorbidity patterns in relation to these associated factors, considering indicators that exhibited statistical significance in the univariate analyses. In the sensitivity analysis, we additionally performed binomial logistic regression to test the aforementioned associated factors in relation to different multimorbidity patterns. Both in terms of multimorbidity pattern identification and associated factor analysis, we can achieve more than 80 % power in our sample. All P-values were two-tailed, and those <0.05 were considered statistically significant. Analyses were performed using R 4.4.0, with the “poLCA” package to perform LCA models.

## 3 Results

### 3.1 Assessment of multimorbidity patterns

**Fig. 2:**
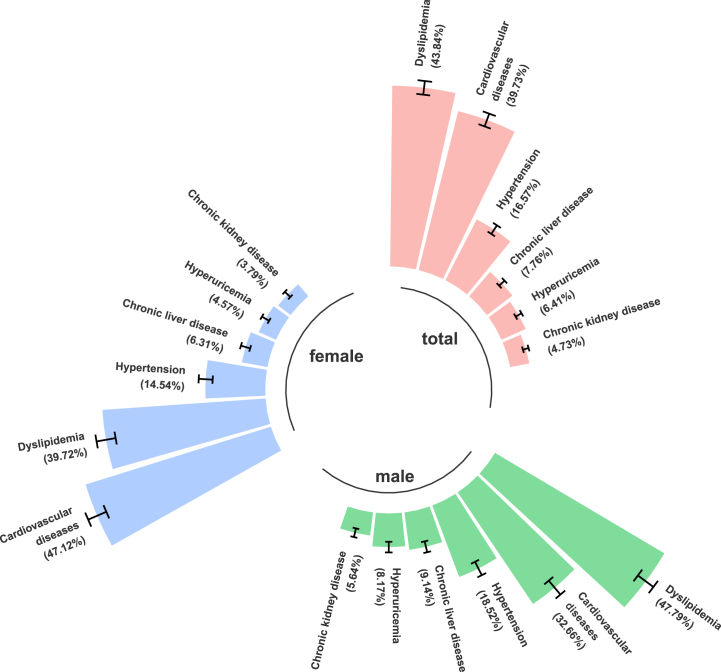
The prevalence of chronic diseases.

A total of 3403 patients with diabetes multimorbidity were included in LCA models, including 1739 (51.2 %) males and 1664 (48.8 %) females. The prevalence of these chronic diseases is shown in Fig. 2, among which the top three multimorbidities are dyslipidemia (with a prevalence of 43.84 %), cardiovascular disease (39.73 %), and hypertension (16.57 %). The chronic condition with the highest prevalence was dyslipidemia with 1492 patients. The model containing 1–4 potential categories (the baseline model) was first estimated, and the fitting statistics are shown in Supplementary Table 1. Taking the minimum AIC as the standard, the three-classification Entropy value should be selected as an acceptable range. Considering all indicators comprehensively and considering the simplicity of the model, the three-classification model should be selected as the ideal model.

The latent class probabilities in the best model (ClASS3) were 0.37, 0.47, and 0.16, with a total of 1. The 3-classification model, exhibits a minimum AIC value of 14396.29 and a BIC value of 14518.94 (Supplementary Table 2), indicating that 37 % of the subjects were classified into T2DM with cardiovascular diseases (Class1), 47 % into T2DM with dyslipidemia (Class2), and 16 % into T2DM with hypertension (Class3). Together with a group of patients without any comorbidities, a total of four multimorbidity patterns were ultimately identified. The distribution of chronic conditions in each pattern is shown in Supplementary Fig. 1.

### 3.2 Associated factors analysis of multimorbidity patterns

A total of 1779 T2DM patients with baseline information were included in this part of the analysis, including 807 (45.4 %) females. Among them, 301 patients (16.9 %) had multimorbidity. The number of subjects using oral hypoglycemic agents was 1771 (99.6 %), and the number of subjects treated with insulin was 1564 (87.9 %). The differences in demographic characteristics, daily living habits, and treatment among the three multimorbidity patterns are shown in Table 1.

**Table 1:** Baseline characteristics of patients with different multimorbidity patterns.

Characteristics	Overall (N = 1779)	Multimorbidity-free (n = 1186)	Cardiovascular diseases (n = 92)	Dyslipidemia (n = 148)	Hypertension (n = 353)	P-value
**Demographics**						
Female, n (%)	807 (45.4)	551 (46.5)	38(41.3)	86 (58.1)	132 (37.4)	<0.001
Age, n (%)						0.003
<60	1201 (67.5)	802 (67.6)	56(60.9)	85 (57.4)	258 (73.1)	
≥60	578 (32.5)	384 (32.4)	36(39.1)	63 (42.6)	95 (26.9)	
Smoker, n (%)						0.005
Non-smoker	713 (40.1)	468 (39.5)	41(44.6)	43 (29.1)	161 (45.6)	
Smoker	1066 (59.9)	718 (60.5)	51(55.4)	105 (70.9)	192 (54.4)	
Drinker, n (%)						0.011
Non-drinker	642 (36.1)	423 (35.7)	36(39.1)	38 (25.7)	145 (41.1)	
Drinker	1137 (63.9)	763 (64.3)	56(60.9)	110 (74.3)	208 (58.9)	
**Clinical characteristics**						
Family history, n (%)	1384 (77.8)	937 (79.0)	67(72.8)	121 (81.8)	259 (73.4)	0.053
BMI, n (%)						0.001
<25	638 (35.9)	437 (36.8)	21(22.8)	65 (43.9)	115 (32.6)	
25-30	800 (45.0)	539 (45.4)	47(51.1)	64 (43.2)	150 (42.5)	
≥30	341 (19.2)	210 (17.7)	24(26.1)	19 (12.8)	88 (24.9)	
Hba1c, n (%)						0.140
<7	352 (19.8)	223 (18.8)	26(28.3)	33 (22.3)	70 (19.8)	
≥7	1427 (80.2)	963 (81.2)	66(71.7)	115 (77.7)	283 (80.2)	
TC, mean (SD)	5.52 (1.5)	5.41 (1.4)	5.53 (1.5)	5.28 (1.3)	6.02 (1.8)	<0.001
TG, mean (SD)	2.63 (3.2)	2.37 (2.4)	2.37 (2.2)	2.11 (2.1)	3.77 (5.33)	<0.001
HDL, mean (SD)	1.22 (0.3)	1.22 (0.3)	1.20 (0.3)	1.33 (0.3)	1.18 (0.3)	<0.001
LDL, mean (SD)	3.59 (1.0)	3.53 (1.0)	3.68 (1.11)	3.33 (1.0)	3.88 (1.1)	<0.001
Blood glucose, mean (SD)	10.33 (3.9)	10.27 (4.0)	9.38 (3.2)	10.16 (3.6)	10.84 (3.9)	0.008
**Treatment patterns**						
Antidiabetic use, n (%)	1771 (99.6)	1183 (99.7)	92 (100.0)	147 (99.3)	349 (98.9)	0.152
Insulin use, n (%)	1564 (87.9)	1055 (89.0)	72(78.3)	144 (97.3)	293 (83.0)	<0.001

Abbreviations: BMI = body mass index, SBP = systolic blood pressure, DBP = diastolic blood pressure, HbA1c = glycosylated hemoglobin, TC = total cholesterol, TG = triglyceride, HDL = high-density lipoprotein cholesterol, LDL = low-density lipoprotein cholesterol, SD = standard deviation.

Multi-nominal logistic regression analysis was used to investigate the factors affecting multimorbidity patterns of T2DM, with multimorbidity-free as reference categories (Table 2). The univariate analysis of each variable is shown in Supplementary Table 4. In multivariate analysis, compared with the multimorbidity-free, patients with cardiovascular disease patterns (Class1) had greater BMI (OR [95 % CI]: 2.52 [1.35,4.70]) and LDL (OR [95 % CI]: 1.27 [1.00,1.60]), and were more likely not to be treated with insulin (OR [95 % CI]: 2.19 [1.29,3.73). Patients with dyslipidemia (Class2) had older age (OR [95 % CI]: 1.45 [1.02,2.08), higher HDL (OR [95 % CI]: 2.95 [1.67,5.21]), lower LDL (OR [95 % CI]: 0.71 [0.59,0.87]), and were more likely to be treated with insulin (OR [95 % CI]: 0.23 [0.08,0.63]). Patients with hypertension (Class 3) were more likely to be male (OR [95 % CI]: 0.73 [0.56,0.95]), had higher TG (OR [95 % CI]: 1.09 [1.04,1.13]) and LDL (OR [95 % CI]: 1.39 [1.22,1.58]), and were more likely not to be treated with insulin (OR [95 % CI]: 1.78 [1.26,2.50]). In the sensitivity analysis conducted via binomial logistic regression, the aforementioned tested factors were also associated with the corresponding multimorbidity patterns.

**Table 2:** Multinomial logistic regression analyses of multimorbidity patterns (reference: multimorbidity-free patterns).

Factors	Levels	Cardiovascular diseases		Dyslipidemia		Hypertension	
		OR (95 % CI)	P-value	OR (95 % CI)	P-value	OR (95 % CI)	P-value
Sex	Male	REF		REF		REF	
	Female	0.80(0.51,1.27)	0.350	1.36(0.94,1.95)	0.101	0.73(0.56,0.95)	0.020
Age	<60	REF		REF		REF	
	≥60	1.47(0.94,2.31)	0.095	1.45(1.02,2.08)	0.040	0.91(0.69,1.21)	0.523
BMI	<25	REF		REF		REF	
	25–30	1.85(1.08,3.17)	0.025	0.89(0.61,1.29)	0.529	0.96(0.72,1.28)	0.778
	≥30	2.52(1.35,4.70)	0.004	0.71(0.41,1.23)	0.225	1.39(0.99,1.96)	0.055
TG	Continuous	0.97(0.87,1.08)	0.537	1.03(0.94,1.13)	0.495	1.09(1.04,1.13)	<0.001
HDL	Continuous	0.70(0.29,1.67)	0.419	2.95(1.67,5.21)	<0.001	0.73(0.45,1.20)	0.217
LDL	Continuous	1.27(1.00,1.60)	0.048	0.71(0.59,0.87)	<0.001	1.39(1.22,1.58)	<0.001
Insulin use	Yes	REF		REF		REF	
	No	2.19(1.29,3.73)	0.004	0.23(0.08,0.63)	0.004	1.78(1.26,2.50)	<0.001

Abbreviations: BMI = body mass index, CI = confidence interval, OR = odds ratio, TG = triglyceride, HDL = high-density lipoprotein cholesterol, LDL = low-density lipoprotein cholesterol, REF = reference.

## 4 Discussion

In this population-based retrospective observational study of T2DM patients, we found that dyslipidemia, cardiovascular diseases, and hypertension were the more common chronic diseases among people with T2DM, and 16.9 % of T2DM patients suffered from multimorbidity. In addition, we also found four different patterns of T2DM-related multimorbidity, including multimorbidity-free, T2DM with cardiovascular diseases mainly, T2DM with dyslipidemia mainly, and T2DM with hypertension mainly. These patterns have different associated factors, including sex, age, BMI, TG, HDL, LDL, and insulin use.

Previous studies on the multimorbidity status of T2DM have mostly been conducted in high-income countries, and the prevalence of comorbidities was highly heterogeneous between studies [8,12,14,[17], [18], [19]]. Hypertension, cardiometabolic precursor conditions (e.g., dyslipidemia, obesity, and hypertension), and cardiovascular diseases, which are consistent with the mechanism of diabetes, are the core of T2DM-related multimorbidity [20]. In this study, we found that the top three comorbidities with the highest prevalence of T2DM were dyslipidemia, cardiovascular diseases, and hypertension in Tianjin, which was basically the same trend as previous studies [12,18,19]. However, the prevalence of hypertension was the first in most studies [8,14,18], but dyslipidemia was present in this population, suggesting that there may be group differences in T2DM-related comorbidities.

Currently, there is no universally accepted measure of multimorbidity. The latent category analysis not only considers the number (burden) of comorbidities but also considers the complex correlation between various chronic diseases and further divides them into different multimorbidity modes, providing a more comprehensive multimorbidity evaluation method [13]. A review summarized seven studies that used cluster analysis to explore multimorbidity patterns in T2DM before 2020 and found that cardiometabolic precursor conditions, vascular conditions, and mental health conditions were the three most common multimorbidity patterns [13]. A hospital-based population-based study from Peru identified four multimorbidity clusters of T2DM (T2DM with no other chronic disease, T2DM with obesity only, T2DM with hypertension but without obesity, and T2DM with all other chronic conditions) [15], while another Basque study identified as many as 10 clusters of multimorbidity [12]. Our results identified four common multimorbidity patterns, including multimorbidity-free, T2DM with cardiovascular diseases, T2DM with dyslipidemia, and T2DM with hypertension. Direct comparisons of multimorbidity patterns across different studies are complicated by considerable heterogeneity in methods and definitions [21]. However, this study used as accurate data and a reasonable method as possible to identify the multimorbidity patterns of T2DM so that our findings can be interpreted in the context of our stated goals, which can provide information on frequently occurring disease combinations for clinical management, as well as non-random disease associations that can indicate common disease mechanisms [22].

Limited studies have further investigated the specific contributing factors of different multimorbidity patterns in patients with T2DM, as most studies have focused on factors influencing comorbidity burden [23]. Older age and higher deprivation have been proven to be associated with T2DM-related multimorbidity [13]. The association of T2DM-associated multimorbidity clustering with common physiological risk factors associated with complications of T2DM, such as HbA1c, blood pressure, and lipids, has not been examined [13]. We found that LDL and insulin use were associated with all multimorbidity patterns, while BMI was associated with multimorbidity clusters dominated by cardiovascular diseases; age and HDL were associated with multimorbidity clusters dominated by dyslipidemia; sex and TG were associated with multimorbidity clusters dominated by hypertension. These different associated factor profiles may suggest that the progression of comorbidities in T2DM can be predicted in the early stages after the diagnosis of T2DM through the patient's individualized characteristics, thus providing evidence for reducing the burden of comorbidities.

### 4.1 Strengths and limitations

Strengths of this study include the use of LCA to identify multimorbidity patterns in patients with T2DM and further characterize the specific associated factors of different patterns for predictive purposes. Nonetheless, some limitations should be pointed out. First, the diagnosis of chronic diseases was based on the inpatient registry system, we could only consider the mainly specific chronic diseases recorded in the plan, and cannot consider all possible comorbidities of chronic diseases. Second, one prominent limitation of this study is the potential selection bias resulting from missing data. Consequently, the sample ultimately analyzed might not comprehensively represent the entire target population, which could potentially overestimate or underestimate the associations between associated factors and multimorbidity patterns, thereby affecting the representativeness of the results. Third, due to the cross-sectional nature of this study, our findings cannot determine the directionality or causality of these associations. In addition, the hospital selected for this study is the largest metabolic disease hospital in Tianjin and basically encompasses all the diabetic patients in Tianjin. However, due to the single-center nature of our research, caution is required in interpreting the specific associated factors of these different multimorbidity patterns found in this study. Future large-sample, multi-regional longitudinal studies may help to elucidate the multimorbidity patterns and their specific associated factors in patients with T2DM, and further explore the impact of these multimorbidity patterns on the prognosis of T2DM. Finally, due to the unavailability of data on the treatment of diabetes and other diseases, it is challenging for us to assess the influence of these clinical treatments on the findings, thereby resulting in potential residual confounding that may affect the results.

## 5 Conclusion

Using an LCA model, we found that there were four distinct multimorbidity patterns among patients with T2DM, including multimorbidity-free, T2DM with cardiovascular diseases mainly, T2DM with dyslipidemia mainly, and T2DM with hypertension mainly. Also, the associated factors of these multimorbidity patterns are different. In treating T2DM, identifying multimorbidity patterns is crucial. Physicians can select hypoglycemic drugs more precisely to avoid exacerbating comorbidity risks. Moreover, personalized nursing plans can be formulated since medical staff can predict problems, allocate resources rationally, improve the quality of nursing care, and enhance patient satisfaction. For diagnosed patients, understanding comorbidity patterns helps to develop comprehensive health management plans, including regular screening and personalized education. This enables patients to manage their conditions better and reduces the consumption of medical resources and the social and economic burdens caused by disease progression.

## CRediT authorship contribution statement

**Jin Li:** Writing – review & editing, Writing – original draft, Methodology, Formal analysis. **Hou Hou:** Validation, Software, Methodology. **Yong Zhang:** Writing – review & editing, Data curation. **Jing Li:** Writing – review & editing, Supervision, Resources, Project administration, Funding acquisition.

## Ethics/ethical approval

The research protocol was approved by the Ethics Committee of Chu Hsien-I Memorial Hospital (ZXYJNYYKMEC2023-28). Since patient health records are anonymous and confirmed prior to analysis, informed consent was not obtained.

## Data availability

The datasets generated during and analyzed during the current study are available

From the corresponding author on reasonable request.

## Study funding

This work was supported by the Tianjin Medical Key Discipline (Specialist) Construction Project (grant number TJYXZDXK-032A) and the Tianjin Metabolic Chronic Disease Prevention and Control System Construction Project (grant number 2019JWZD54).The founder had no role in study design, data collection, data analysis, data interpretation, or writing of the report. The corresponding author had full access to the data in the study and had final responsibility for the decision to submit for publication.

## Declaration of competing interest

The authors declare that they have no known competing financial interests or personal relationships that could have appeared to influence the work reported in this paper.

## References

[1] Chen X., Giles J., Yao Y. (2022). The path to healthy ageing in China: a Peking University-Lancet Commission. Lancet.

[2] Fu Y., Chen M., Si L. (2022). Multimorbidity and catastrophic health expenditure among patients with diabetes in China: a nationwide population-based study. BMJ Glob. Health.

[3] EG (2019). Global burden of disease study 2019 (GBD 2019) data resources. http://ghdx.healthdata.org/gbd-results-tool.

[4] Wang L., Gao P., Zhang M. (2017). Prevalence and ethnic pattern of diabetes and prediabetes in China in 2013. JAMA.

[5] Collaborators G.B.D.N. (2019). Global, regional, and national burden of neurological disorders, 1990-2016: a systematic analysis for the Global Burden of Disease Study 2016. Lancet Neurol..

[6] Dalys G.B.D., Collaborators H., Murray C.J. (2015). Global, regional, and national disability-adjusted life years (DALYs) for 306 diseases and injuries and healthy life expectancy (HALE) for 188 countries, 1990-2013: quantifying the epidemiological transition. Lancet.

[7] (WHO) WHO. Noncommunicable diseases. https://www.who.int/news-room/fact-sheets/detail/noncommunicable-diseases(05.31 2024).

[8] Teljeur C., Smith S.M., Paul G., Kelly A., O'Dowd T. (2013). Multimorbidity in a cohort of patients with type 2 diabetes. Eur. J. Gen. Pract..

[9] Collaborators G.B.D.M. (2018). Global, regional, and national age-sex-specific mortality and life expectancy, 1950-2017: a systematic analysis for the Global Burden of Disease Study 2017. Lancet.

[10] Jamison D.T., Summers L.H., Alleyne G. (2013). Global health 2035: a world converging within a generation. Lancet.

[11] Zhang Z., He P., Yao H. (2023). A network-based study reveals multimorbidity patterns in people with type 2 diabetes. iScience.

[12] Alonso-Morán E., Orueta J.F., Esteban J.I. (2015). Multimorbidity in people with type 2 diabetes in the Basque Country (Spain): prevalence, comorbidity clusters and comparison with other chronic patients. Eur. J. Intern. Med..

[13] Cicek M., Buckley J., Pearson-Stuttard J., Gregg E.W. (2021). Characterizing multimorbidity from type 2 diabetes: insights from clustering approaches. Endocrinol Metab. Clin. N. Am..

[14] Soji D.J., Lordson J., Mini G.K. (2021). Multimorbidity patterns among rural adults with Type-2 diabetes mellitus: a cross-sectional study in Kerala, India. WHO South East Asia J Public Health.

[15] Bernabe-Ortiz A., Borjas-Cavero D.B., Páucar-Alfaro J.D., Carrillo-Larco R.M. (2022). Multimorbidity patterns among people with type 2 diabetes mellitus: findings from Lima, Peru. Int. J. Environ. Res. Publ. Health.

[16] Nichols L., Taverner T., Crowe F. (2022). In simulated data and health records, latent class analysis was the optimum multimorbidity clustering algorithm. J. Clin. Epidemiol..

[17] Chen Y., Pan M., He Y. (2023). Disease burden and the accumulation of multimorbidity of noncommunicable diseases in a rural population in henan, China: cross-sectional study. JMIR Public Health Surveill.

[18] Chiang J.I., Hanlon P., Li T.C. (2020). Multimorbidity, mortality, and HbA1c in type 2 diabetes: a cohort study with UK and Taiwanese cohorts. PLoS Med..

[19] Nowakowska M., Zghebi S.S., Ashcroft D.M. (2019). The comorbidity burden of type 2 diabetes mellitus: patterns, clusters and predictions from a large English primary care cohort. BMC Med..

[20] Aguado A., Moratalla-Navarro F., López-Simarro F., Moreno V. (2020). MorbiNet: multimorbidity networks in adult general population. Analysis of type 2 diabetes mellitus comorbidity. Sci. Rep..

[21] TaoM Sciences Multiple long-term conditions (multimorbidity): a priority for global health research. https://acmedsci.ac.uk/policy/policy-projects/multimorbidity.

[22] Whitty C.J.M., Watt F.M. (2020). Map clusters of diseases to tackle multimorbidity. Nature.

[23] Gao F., Chen J., Liu X. (2017). Latent class analysis suggests four classes of persons with type 2 diabetes mellitus based on complications and comorbidities in Tianjin, China: a cross-sectional analysis. Endocr. J..

