# Supplementary material for: Multimorbidity patterns and their associated factors among patients with type 2 diabetes in China: A hospital-based observational study
Source: Heliyon. 2025 Feb 21;11(4):e42905. doi: 10.1016/j.heliyon.2025.e42905 (PMC11903810; doi:10.1016/j.heliyon.2025.e42905)
Supplement: Multimedia component 1 [file mmc1.docx]

**Supplementary Table 1.** Fit indices for latent class modeling

| Model | AIC | BIC | Maximum log-likelihood | G2 | Entropy |
| --- | --- | --- | --- | --- | --- |
| CLASS1 | 17080.38 | 17117.18 | -8534.19 | 3680.19 | 2.51 |
| CLASS2 | 14943.72 | 15023.44 | -7458.86 | 1529.53 | 2.19 |
| CLASS3 | 14396.29 | 14518.94 | -7178.14 | 968.10 | 2.11 |
| CLASS4 | 14696.53 | 14862.11 | -7321.27 | 1254.34 | 2.15 |

Note: The selection of the final multimorbidity pattern model was made by simultaneously considering the minimum Akaike Information Criterion (AIC), Bayesian Information Criterion (BIC), maximum log-likelihood, G2, and entropy. The model with three latent variables complied with almost all of the above-mentioned criteria.

**Supplementary Table 2.** Conditional probability of LCA model.

| **Class name** | **Disease condition (Item response probabilities)** | | | | | | **Membership probabilities** |
| --- | --- | --- | --- | --- | --- | --- | --- |
|  | **Dyslipidemia** | **Hypertension** | **Hyperuricemia** | **Cardiovascular diseases** | **Chronic kidney disease** | **Chronic liver disease** |  |
| Cardiovascular diseases | 0.03 | 0.01 | 0.00 | 1.00 | 0.01 | 0.00 | 0.37 |
| Dyslipidemia | 0.80 | 0.04 | 0.13 | 0.01 | 0.07 | 0.15 | 0.47 |
| Hypertension | 0.20 | 1.00 | 0.01 | 0.19 | 0.05 | 0.01 | 0.16 |

Probabilities >50% are bolded

Class names are based on either the disease/s with the highest probability or categories of clinical significance


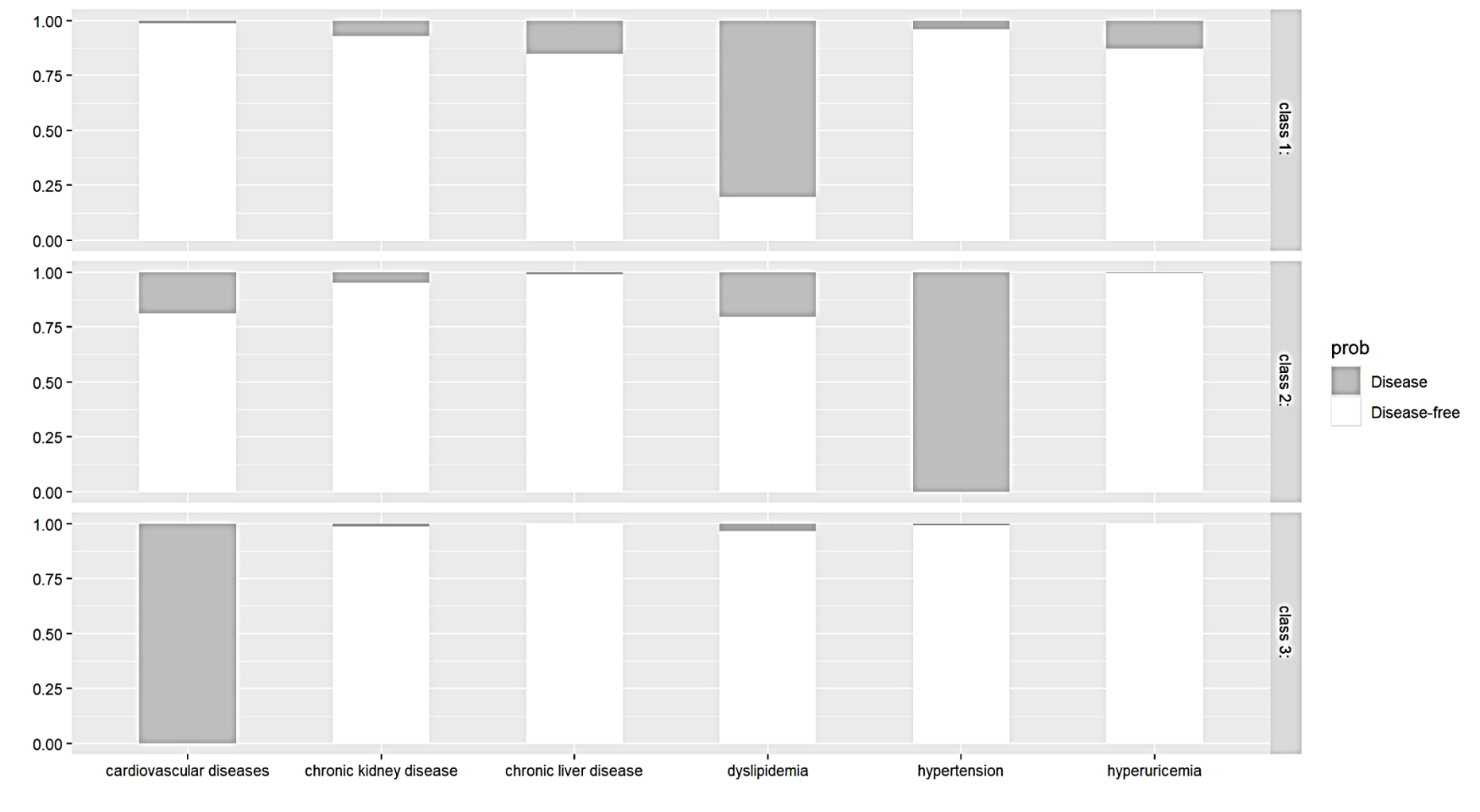


**Supplementary Figure 1**. The conditional probability of each multimorbidity class.

**Supplementary Table 3** Baseline characteristics of patients.

| **Characteristics** | **Overall** | **Included in the associated factors analysis** |
| --- | --- | --- |
|  | **(N=3403)** | **(N=1779)** |
| Gender, n (%) |  |  |
| Female | 1664 (48.9) | 807 (45.4) |
| Male | 1739 (51.1) | 972 (54.6) |
| Age, n (%) |  |  |
| <60 | 2240 (65.8) | 1201 (67.5) |
| ≥60 | 1163 (34.2) | 578 (32.5) |
| Hba1c, n (%) |  |  |
| <7 | 1106 (32.5) | 352 (19.8) |
| ≥7 | 2297 (67.5) | 1427 (80.2) |
| TC, mean (SD) | 5.53 (1.4) | 5.52 (1.5) |
| TG, mean (SD) | 2.42 (2.9) | 2.63 (3.2) |
| HDL, mean (SD) | 1.30 (0.3) | 1.22 (0.3) |
| LDL, mean (SD) | 3.56 (1.0) | 3.59 (1.0) |
| Blood glucose, mean (SD) | 9.57 (3.2) | 10.33 (3.9) |
| Antidiabetic use, n (%) | 3378 (99.3) | 1771 (99.6) |
| Insulin use, n (%) | 2406 (70.7) | 1564 (87.9) |

Abbreviations: HbA1c= glycosylated hemoglobin, TC= total cholesterol, TG= triglyceride, HDL= high-density lipoprotein cholesterol, LDL= low-density lipoprotein cholesterol, SD = standard deviation.

**Supplementary Table 4** Multinomial logistic regression analyses of multimorbidity patterns (reference: multimorbidity-free patterns): univariate analysis.

| **Factors** | **Cardiovascular diseases** | | **Dyslipidemia** | | **Hypertension** | |
| --- | --- | --- | --- | --- | --- | --- |
|  | **OR (95% CI)** | **P-value** | **OR (95% CI)** | **P-value** | **OR (95% CI)** | **P-value** |
| Sex |  |  |  |  |  |  |
| Male | REF |  | REF |  | REF |  |
| Female | 0.81 (0.53,1.25) | 0.340 | 1.60 (1.13,2.26) | 0.008 | 0.69 (0.54,0.88) | 0.003 |
| Age |  |  |  |  |  |  |
| <60 | REF |  | REF |  | REF |  |
| ≥60 | 1.34 (0.87,2.08) | 0.185 | 1.55 (1.09,2.19) | 0.014 | 0.77 (0.59,1.00) | 0.052 |
| Smoker |  |  |  |  |  |  |
| Non-smoker | REF |  | REF |  | REF |  |
| Smoker | 1.24 (0.81,1.89) | 0.333 | 0.63 (0.43,0.91) | 0.015 | 1.29 (1.01,1.64) | 0.039 |
| Drinker |  |  |  |  |  |  |
| Non-drinker | REF |  | REF |  | REF |  |
| Drinker | 1.16 (0.75,1.79) | 0.506 | 0.62 (0.42,0.92) | 0.017 | 1.26 (0.99,1.60) | 0.065 |
| Family history | 0.71 (0.44,1.15) | 0.165 | 1.19 (0.77,1.85) | 0.437 | 0.73 (0.56,0.96) | 0.026 |
| BMI |  |  |  |  |  |  |
| <25 | REF |  | REF |  | REF |  |
| 25-30 | 1.81 (1.08,3.17) | 0.028 | 0.80 (0.61,1.29) | 0.230 | 1.06 (0.72,1.28) | 0.689 |
| ≥30 | 2.38 (1.35,4.70) | 0.005 | 0.61 (0.41,1.23) | 0.070 | 1.59 (0.99,1.96) | 0.005 |
| HbA1c |  |  |  |  |  |  |
| <7 | REF |  | REF |  | REF |  |
| ≥7 | 0.59 (0.36,0.95) | 0.029 | 0.81 (0.53,1.22) | 0.310 | 0.94 (0.69,1.26) | 0.666 |
| TC, Continuous | 1.07 (0.92,1.24) | 0.381 | 0.93 (0.82,1.06) | 0.289 | 1.30 (1.20,1.40) | < 0.001 |
| TG, Continuous | 1.00 (0.91,1.10) | 0.987 | 0.93 (0.85,1.03) | 0.170 | 1.12 (1.08,1.16) | < 0.001 |
| HDL, Continuous | 0.78 (0.38,1.61) | 0.510 | 2.73 (1.65,4.53) | < 0.001 | 0.67 (0.44,1.00) | 0.051 |
| LDL, Continuous | 1.16 (0.94,1.42) | 0.161 | 0.81 (0.67,0.97) | 0.021 | 1.37 (1.22,1.53) | < 0.001 |
| Blood glucose, Continuous | 0.93 (0.88,0.99) | 0.034 | 0.99 (0.95,1.04) | 0.754 | 1.04 (1.01,1.07) | < 0.018 |
| Antidiabetic use |  |  |  |  |  |  |
| Yes | REF |  | REF |  | REF |  |
| No | 0.02 (0.88,0.99) | 0.795 | 2.64 (0.95,1.04) | 0.404 | 4.52 (1.01,1.07) | 0.049 |
| Insulin use |  |  |  |  |  |  |
| Yes | REF |  | REF |  | REF |  |
| No | 2.24 (1.32,3.79) | 0.003 | 0.22 (0.08,0.61) | 0.004 | 1.65 (1.18,2.30) | 0.003 |

Abbreviations: BMI= body mass index, CI= confidence interval, OR= odds ratio, TC= total cholesterol, TG= triglyceride, HbA1c= glycosylated hemoglobin, HDL= high-density lipoprotein cholesterol, LDL= low-density lipoprotein cholesterol, REF= reference.

**Supplementary Table 5** Binomial logistic regression analyses of multimorbidity patterns (reference: multimorbidity-free patterns): sensitivity analysis.

| Factors | Levels | Cardiovascular diseases | | Dyslipidemia | | Hypertension | |
| --- | --- | --- | --- | --- | --- | --- | --- |
|  |  | OR (95% CI) | P-value | OR (95% CI) | P-value | OR (95% CI) | P-value |
| Sex | Male | REF |  | REF |  | REF |  |
|  | Female | 0.81(0.51,1.28) | 0.373 | 1.36(0.95,1.97) | 0.095 | 0.72(0.55,0.94) | 0.014 |
| Age | <60 | REF |  | REF |  | REF |  |
|  | ≥60 | 1.47(0.93,2.31) | 0.096 | 1.48(1.03,2.12) | 0.033 | 0.90(0.68,1.19) | 0.464 |
| BMI | <25 | REF |  | REF |  | REF |  |
|  | 25-30 | 1.84(1.07,3.16) | 0.026 | 0.90(0.62,1.31) | 0.580 | 0.96(0.72,1.28) | 0.783 |
|  | ≥30 | 2.47(1.32,4.62) | 0.005 | 0.72(0.41,1.24) | 0.235 | 1.40(0.99,1.96) | 0.054 |
| TG | Continuous | 0.97(0.87,1.07) | 0.506 | 1.03(0.95,1.12) | 0.494 | 1.09(1.04,1.14) | < 0.001 |
| HDL | Continuous | 0.74(0.31,1.75) | 0.488 | 2.97(1.68,5.26) | < 0.001 | 0.77(0.47,1.26) | 0.306 |
| LDL | Continuous | 1.25(0.99,1.57) | 0.058 | 0.73(0.60,0.88) | 0.001 | 1.38(1.21,1.58) | < 0.001 |
| Insulin use | Yes | REF |  | REF |  | REF |  |
|  | No | 2.15(1.26,3.67) | 0.004 | 0.22(0.08,0.61) | 0.004 | 1.82(1.29,2.56) | < 0.001 |

Abbreviations: BMI= body mass index, CI= confidence interval, OR= odds ratio, TG= triglyceride, HDL= high-density lipoprotein cholesterol, LDL= low-density lipoprotein cholesterol, REF= reference.
